# Rebuilding the gut: Decoding enteric pathogenesis using next-generation human *in vitro* models

**DOI:** 10.1371/journal.ppat.1014718

**Published:** 2026-10-26

**Authors:** Linus H. Schweizer, Ashley Gilliland, Yan Chen, Artemis Melville-Bowser, Bruce A. Vallance

**Affiliations:** Department of Pediatrics, BC Children’s Hospital, University of British Columbia, Vancouver, British Columbia, Canada; Mater Research Institute - University of Queensland, AUSTRALIA

## Abstract

Traditional *in vitro* and *in vivo* models often fail to replicate the complex physiology of the human gastrointestinal (GI) tract, and consequently, do not fully capture how this environment influences the infective strategies employed by enteric pathogens. Here, we evaluate air-liquid interface (ALI) cultures and gut-on-a-chip (GoC) microfluidic systems as next-generation *in vitro* models (NGIMs) that recreate critical features of the human GI mucosal surface, including the mucus layer, crypt-villus architecture, and biomechanical forces that modulate enteric pathogenesis. We review their application towards bacterial, fungal, viral, and parasitic infections, highlighting novel mechanisms that were previously undetected using traditional models. Comparing the two approaches, we outline how ALI excels for studies involving mucus and for cost-effective implementation, whereas GoC systems uniquely capture peristalsis and luminal flow, revealing dynamic pathogen-host interactions. Notably, both platforms enable complex co-culturing, allowing pathogen-host-commensal dynamics to be studied in a reductionist manner. Lastly, we discuss how future efforts aimed at better mimicking the human GI tract could incorporate stromal, immune, and enteric nervous system components, while also improving accessibility and standardization. Taken together, both ALI and GoC systems offer significant opportunities to provide new insights into infection biology, while also serving as effective preclinical platforms for precision medicine and the evaluation of next-generation antimicrobial therapies.

## Introduction

The gastrointestinal (GI) tract serves as a critical interface between the host and its external environment, making it a target for diverse food and water-borne pathogens, including bacteria, fungi, viruses, and parasites. Enteric pathogens impose major health burdens across the world and are a significant cause of mortality, particularly in children and other vulnerable populations [1,2]. Despite the field’s history of advances in the management of enteric infections, the rise of antimicrobial resistance (AMR) in clinically important enteric pathogens, including *Shigella* species, *Candida* species, and *Clostridioides difficile*, highlights the growing need to develop new anti-infection strategies [3,4]. Beyond their acute effects, GI infections have also been linked to chronic conditions including post-infectious irritable bowel syndrome (PI-IBS) and inflammatory bowel disease (IBD), underscoring the importance of preventing and treating enteric infections [5,6].

Traditional models, such as cell lines, 3D organoids, and animal species, have clearly helped define the virulence mechanisms used by enteric pathogens, but unfortunately, they have several limitations. Unlike the human intestinal mucosa, which is protected by a thick mucus layer, most widely used *in vitro* cell lines such as Caco-2 cells secrete little if any mucus, and HT29-MTX cells only secrete mucins characteristic of the gastric and respiratory mucosa [7,8]. In addition, many clinically important pathogens exhibit host tropism and do not replicate human disease phenotypes in rodent models [9]. For example, human norovirus fails to establish infection in mice, whereas ETEC infection in mice fails to reproduce the characteristic diarrheal symptoms seen in humans [10,11]. Moreover, animal models can display significant variability between laboratories, often due to differences in gut microbiota composition and function [12]. As such, there is a clear need for new and reproducible modeling systems that better mimic the human GI tract.

Intestinal tissue-derived organoids are a promising alternative, especially as the FDA Modernization Act 2.0 and the National Institutes of Health (NIH) push towards the reduction of animal use and advancement of human-based new approach methodologies (NAMs) [13]. These three-dimensional (3D) models have become an attractive platform for studying enteric pathogens; however, they remain challenging to infect because their apical surface and associated features, such as mucus, face inward, requiring pathogens to be laboriously micro-injected into the lumens of individual organoids [14,15]. Furthermore, 3D organoid cultures can exhibit considerable heterogeneity in size and cellular composition, even within a single experiment. These limitations have led to the development of novel organoid-based strategies to more accurately model the native human intestine, in a manner readily accessible for infections.

Advances in bioengineering have led to the air-liquid interface (ALI) and gut-on-a-chip (GoC) culture models, which we have collectively termed next-generation *in vitro* models (NGIMs). Intestinal ALI cultures use Transwell inserts, where intestinal epithelial cell (IEC) monolayers are supplied with media basolaterally, while their apical side is exposed to air [16–18] (Fig 1). This results in the development of crypt-like structures, rapid IEC differentiation into goblet cells, mucin release, and the formation of an overlying mucus layer. In contrast, microfluidic GoC systems are composed of distinct microchannels separated by a porous membrane [19] (Fig 1). The top channel typically contains IECs, while the bottom is seeded with endothelial cells [20]. Like ALI, IECs grown in GoC systems display cellular heterogeneity, crypt-like architecture, and mucus layer development, overcoming many limitations of traditional cell culture. Notably, NGIMs have been adapted to support an apical anaerobic compartment, enabling the study of pathogen-host dynamics involving obligate anaerobic pathogens and commensal microbiota [21,22]. This review will highlight the strengths and weaknesses of the ALI and GoC systems, and the discoveries surrounding enteric pathogens made possible by their use. It will also propose future directions for these platforms to more accurately study microbe-host interactions, as well as their potential to rapidly advance our mechanistic understanding of enteric pathogenesis and facilitate the discovery of new therapeutics.

**Fig 1:**
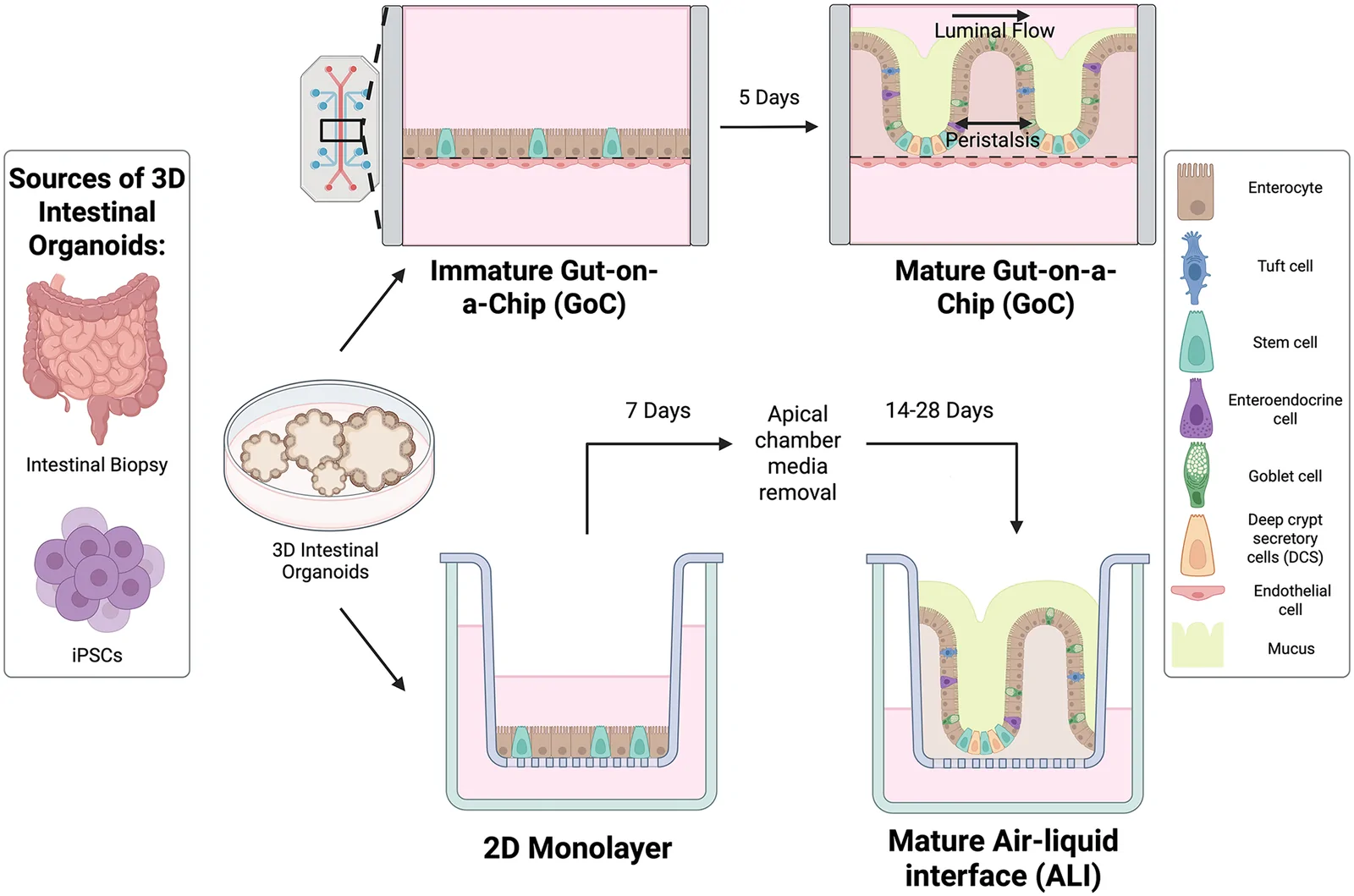
Schematic of Air-Liquid Interface (ALI) and Gut-on-a-Chip (GoC) systems. Human 3D intestinal organoids are derived from intestinal biopsies or induced-pluripotent stem cells (iPSCs). To generate ALI cultures, organoids are dissociated into single cells and seeded onto Transwells. After the monolayer is confluent (~7 days), the apical media is removed, and a mature epithelium develops within 14-28 days. To generate GoC systems, organoids are fragmented and seeded into the upper channel of a porous membrane, while endothelial cells are seeded in the bottom channel. Continuous fluid flow and stretch forces promote maturation of GoC by day 5. Both ALI and GoC systems form a well-differentiated epithelium, a robust mucus layer, and crypt-like architecture. Created in BioRender. Vallance, B. (2026) https://BioRender.com/me5bsrb.

## Modeling understudied aspects of human gut physiology that influence enteric infections

Traditional cell culture, animal, and 3D organoid models are limited in their ability to model several key aspects of human intestinal biology. Here, we describe the importance of these understudied features and how their incorporation into NGIMs has significantly advanced our understanding of enteric infections.

### Gut architecture

Within the GI tract, the crypt-villus axis contains distinct environmental niches that pathogens have evolved to exploit. For example, infections by several pathogens, including *C. difficile* and *Listeria monocytogenes,* have been shown to decrease stem cell-mediated regeneration at the crypt base, thus reducing pathogen clearance from this niche [23,24]. This damage may also increase susceptibility to secondary infections or drive disease recurrence by impairing epithelial repair [24]. Interestingly, the bacterial pathogen *L. monocytogenes* expresses virulence factors to specifically invade villus tips, while *Cryptosporidium* can induce microvilli elongation, potentially supporting parasitic proliferation by enhancing nutrient uptake in infected IECs [25,26]. Unfortunately, traditional human IEC culture methods typically form an IEC monolayer and thus lack these critical intestinal structures [27,28]. In contrast, human-derived NGIMs form crypt-like structures with correct apical-basal polarity, while some also form rudimentary villi and microvilli [16,19,20,29,30]. In GoC systems, 3D villi-like architecture proved necessary for *Shigella flexneri* invasion during early stages of infection, while villi were also critical for infection by viruses like coxsackievirus B1, which preferentially shed from IECs at the apex of infected villi [31,32]. By recapitulating crypt-villus architecture, ALI and GoC systems can capture niche-specific pathogen behavior, elucidating previously unrecognized virulence mechanisms (Fig 2).

**Fig 2:**
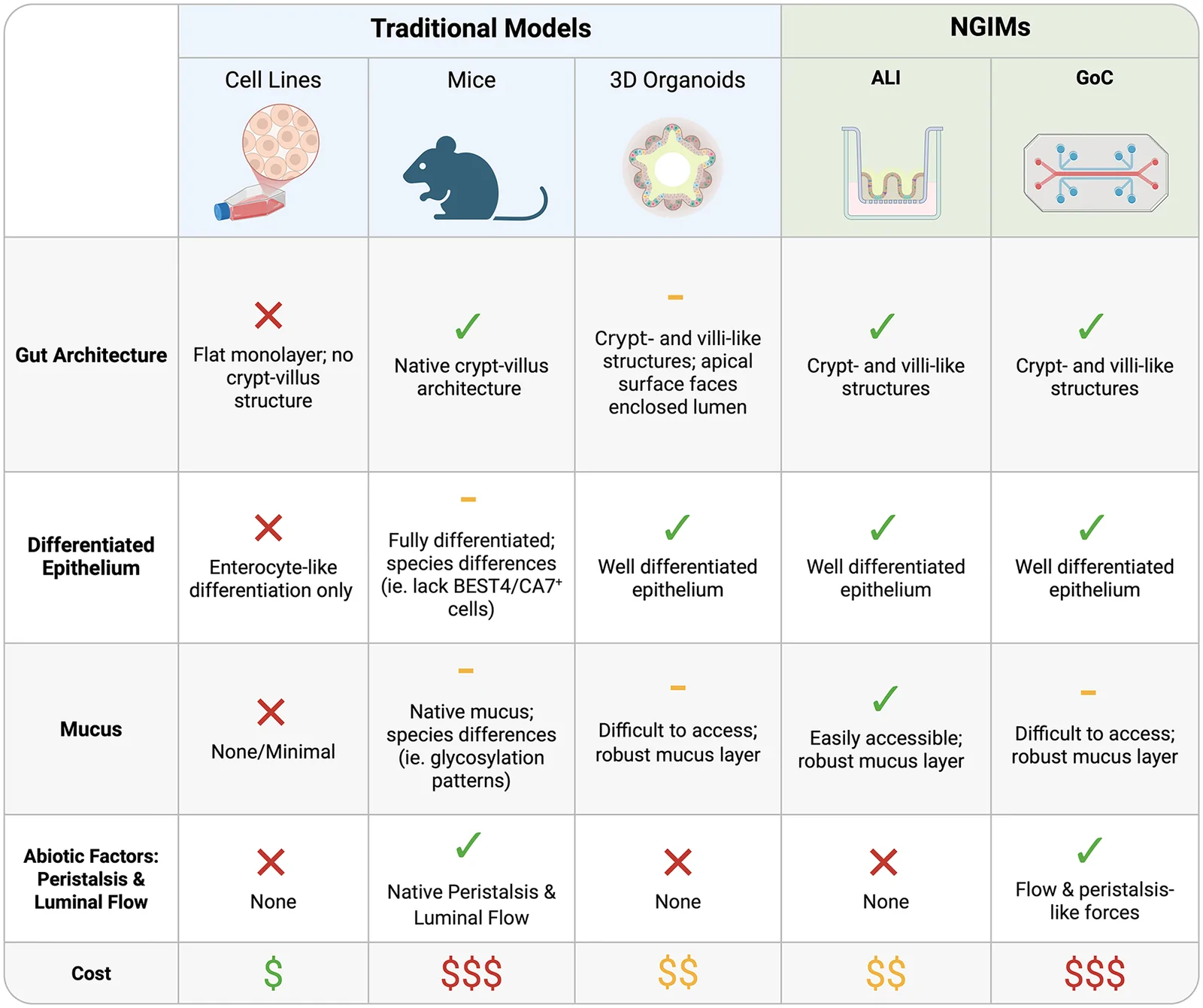
Comparison of ALI, GoC, cell lines, 3D organoids, and mouse models in recapitulating human physiology. The various models are evaluated based on their relative benefits and limitations across key biological characteristics. Green checkmarks indicate strong ability, yellow dashes indicate good ability, and red crosses denote limited ability. NGIMs: Next-generation *in vitro* models. Created in BioRender. Vallance, B. (2026) https://BioRender.com/me5bsrb.

### Differentiated IEC subtypes

The human intestinal epithelium contains multiple IEC subtypes, e.g., goblet cells, Paneth cells, etc., that are key to the regional specificity of the gut. Some of these cells are important for infection modeling, as many enteric pathogens target specific IEC subsets, an aspect of pathogenesis that is easily overlooked when using undifferentiated IEC cultures. Most transformed human IEC lines, including Caco-2 cells, exhibit limited cellular differentiation, underlying the failure of human noroviruses to efficiently replicate in these lines [33]. Similarly, several enteroviruses induce only limited antiviral signaling pathways in these cell lines [34]. Species differences present an additional challenge, as critical target IEC subsets may be absent in animal models. For example, human BEST4/Ca7+ cells were recently identified as targets for heat-stable enterotoxin A (STa), a key diarrhea-inducing toxin produced by enterotoxigenic *Escherichia coli* (ETEC), but these cells are not present in mice [35] (Figs 2 and 3A). In contrast, NGIMs can promote the differentiation of stem cells into all IEC lineages, creating a more physiologically relevant epithelium [16,36]. These platforms can also recapitulate region-specific IEC populations along the small to large intestine axis [20,37]. While 3D organoids can also contain differentiated IEC subtypes, they generally exhibit a less mature phenotype, displaying increased proliferative signatures and reduced transcription of intestinal nutrient transport and metabolism genes when compared to ALI and GoC cultures [20,29,38–40]. These advanced models thus provide a means to investigate how microbes infect the human intestinal epithelium with *in vivo*-like regional and cellular composition, including the presence of rare IEC types that are lacking in traditional models (Fig 2).

**Fig 3:**
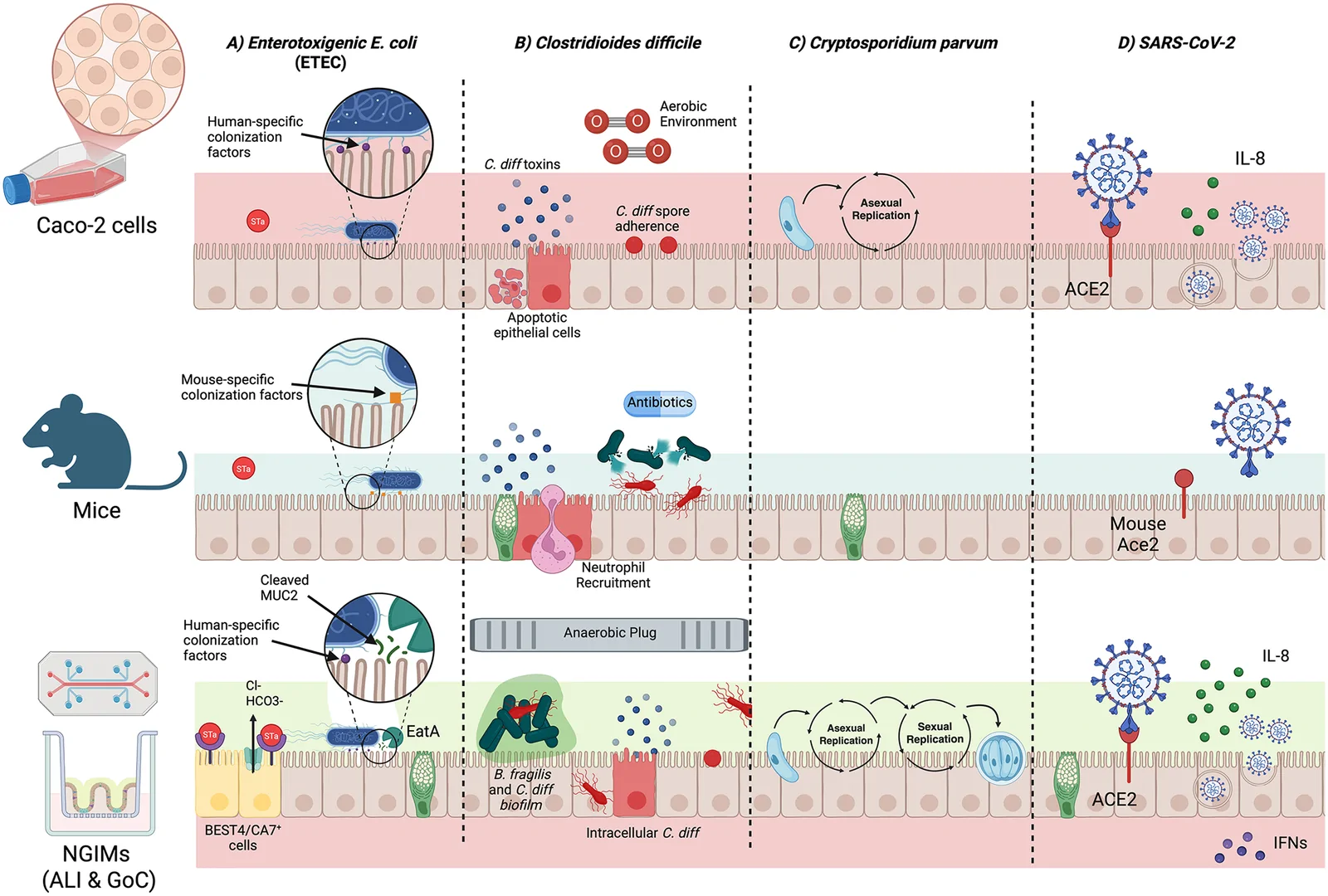
Next-Generation In Vitro Models (NGIMs) are superior to Caco-2 cells and animal models for modeling host-pathogen interactions at the intestinal mucosal surface. Differences in host-pathogen interactions observed across various models are compared. **A)** *Enterotoxigenic Escherichia coli* (ETEC) infection in NGIMs recapitulates human-specific colonization factor interactions, STa-responsive BEST4/CA7+ epithelial cells, and EatA-mediated mucus degradation, features that are absent or limited in conventional cell culture and murine models. **B)** Under anaerobic NGIM conditions, *C. difficile* displays biofilm formation with commensal microbes and shows intracellular infection. In Caco-2 cells, infection is limited due to aerobic culturing conditions, while in mice, although immune activation is present, successful *C. difficile* colonization typically requires antibiotic pre-treatment thereby obscuring the effects of native commensal microbes. **C)** NGIMs support the complete life cycle of *Cryptosporidium parvum,* including sexual reproduction and fertilization, whereas Caco-2 cells do not support these stages, and mice are typically resistant to infection. **D)** SARS-CoV-2 binds inefficiently to murine Ace2, limiting infection in mouse models. In Caco-2 cells, despite receptor compatibility, cytokine production following infection is limited. In contrast, NGIMs elicit a more physiologically relevant and robust cytokine response. Created in BioRender. Vallance, B. (2026) https://BioRender.com/me5bsrb.

### Mucus barrier

Intestinal mucus serves as a critical first line of host defense by physically separating luminal enteric pathogens from the underlying epithelium [41]. Mucus can also influence pathogen behavior, impacting mucinases, virulence, and adherence gene expression [42]. Studies have recently shown that some pathogens have evolved species-specific mechanisms to overcome the intestinal mucus barrier. For example, the EatA serine protease encoded by ETEC can degrade human MUC2, the main polymer of intestinal mucus, *in vitro*, but fails to degrade mouse Muc2 because of species differences in MUC2 structure [43] (Figs 2 and 3A). Thus, key virulence mechanisms may not translate from animal models to humans [43]. Moreover, the thick mucus barrier found in the human colon is comparably thinner in mice, even when considering the size differences between species [44,45]. This difference thus limits the relevance of mouse models when assessing the contributions of mucus to protection against enteric pathogens [45].

Unfortunately, most traditional IEC lines generate little or no mucus [46]. In contrast, IECs cultured in NGIMs display significant goblet cell differentiation and produce a robust mucus layer [47–50] (Fig 2). Notably, ALI monolayers derived from porcine, mouse, and human organoids produce mucus that closely resembles the native species’ GI mucus, as measured using proteomic, glycomic and/or rheologic assays [48,51–53]. GoC systems using human colonic organoids can also form a mucus bilayer that mimics the penetrable outer and impenetrable inner mucus layers seen in the native colon [50]. This development of physiological mucus in ALI and GoC now permits assessment of mucus-mediated protection from diverse pathogenic microbes. In mouse organoid ALI cultures, infection of IECs by the murine pathogen *Citrobacter rodentium* was delayed by the presence of a mucus layer [51]. Similarly, ulcerative colitis patient-derived organoid ALI monolayers showed patient-specific differences in mucus layer development, subsequently impacting their susceptibility to an *E. coli* pathobiont [48]. In GoC systems, *Salmonella enterica* serovar Typhimurium and *E. coli* adherence and invasion were reduced in the presence of mucus, highlighting its role as a physical barrier [54,55]. Taken together, NGIMs produce a physiologically relevant mucus barrier, facilitating research into species-specific colonization strategies along with mucus-dependent pathogenic mechanisms (Fig 2).

### Abiotic factors – anaerobic environment, peristalsis, and fluid flow

In the colon, both IECs and luminal microbes consume oxygen, leading to an anoxic lumen that supports resident obligate anaerobes and a complex microbial community that defends against pathogens through competitive exclusion, nutrient acquisition, and direct killing. Creating an anaerobic environment to support these microbes *in vitro* while keeping host cells oxygenated is challenging using traditional cell culture. Notably, several studies have adapted NGIMs to maintain physiologically relevant oxygen gradients (i.e., oxygenated basolateral compartment with an anaerobic apical compartment), thus allowing co-culture of IECs with an anaerobic gut microbiota for several days [21,22,56,57]. While pathogen-host interactions have yet to be studied using anoxic ALI cultures, in anaerobic GoC systems, co-culture with the commensal anaerobes *Faecalibacterium prausnitzii* or *Bifidobacterium bifidum* was shown to preserve microbial metabolic activity as well as its anti-inflammatory effects on the intestinal epithelium [57,58]. These studies show the potential to study how anoxic conditions impact pathogen virulence, as well as elucidate novel aspects of colonization resistance or host tolerance mechanisms mediated by anaerobic commensals.

Coordinated intestinal muscle contractions, also known as peristalsis, move luminal contents down the digestive tract. Studies using GoC technology incorporating stretch forces that mimic peristalsis-like contractions revealed growth differences between pathogens and commensal microbes *in vitro.* In a GoC model with stretch forces, commensal *E. coli* persistently colonized the system’s lumen without impacting barrier function, whereas enteroinvasive *E. coli* (EIEC) dramatically multiplied at the IEC surface, resulting in villi destruction and impaired barrier function [59]. In addition, under peristalsis-like conditions *S. flexneri* was shown to significantly increase its IEC invasion and intracellular propagation, whereas the parasite *Entamoeba histolytica* displayed slower initial infection of IECs [60]. Despite delayed infection, *E. histolytica* demonstrated increased capacity to disrupt IEC tight junctions and degrade the actin brush border [60]. These results reveal how peristalsis-like forces can influence infection dynamics in GoC systems. However, these findings should be interpreted with caution, as mechanical stretch itself could compromise barrier integrity, independent of its physiological effects on infection, thereby warranting controls to distinguish these effects.

Shear forces generated by luminal contents moving along the length of the GI tract are another important mechanical component. Studies in GoC systems have shown that biomechanical forces like fluid flow influence pathogen infection dynamics by impacting both IEC and pathogen viability *in vitro* [56]. Under flow conditions, *S. flexneri* significantly increased its intracellular propagation and Type 3 secretion system (T3SS) expression [31,60]. *Campylobacter jejuni* infection under flow conditions also induces IEC metabolic reprogramming, characterized by alterations to glycolysis and amino acid metabolism that are absent during static infections [49]. Fluid flow was also shown to extend the *in vitro* culturing time of the enteric parasite *Cryptosporidium hominis,* and influenced the viral infection kinetics of both coxsackievirus B1 and coronaviruses [32,61,62]. In addition, fluid flow appears to enhance the potency of ETEC STa on IEC due to their increased polarization and differentiation [63]. These studies highlight how luminal flow can impact pathogen viability, virulence, and toxin efficacy, and thus may be an important factor to consider when studying enteric pathogen colonization (Fig 2).

## Identifying novel mechanisms of pathogenesis using advanced gut models

NGIMs are regularly being used to uncover pathogenic mechanisms that were previously undetectable using traditional cell culture models or mouse models. Here, we highlight how these models have been used to identify novel aspects of bacterial, fungal, viral, and parasite pathogenesis as well as infection dynamics in complex microbial cultures (Tables 1 and 2).

**Table 1:** Infection modeling using GoC.

Pathogen		Cell type		Major findings	Ref
Domain/phyla	Species	Host species	Source		
Bacteria	*Clostridioides difficile* and *Bacteroides fragilis*	Human	Caco-2 cells	GoC supports long-term co-culture of anaerobic bacteria, *C. difficile* and *B. fragilis*. During vancomycin treatment, *C. difficile* associates with *B. fragilis*, potentially increasing its survival under antibiotic exposure. *C. difficile* is also capable of intracellular invasion of Caco-2 cells grown on GoC.	Bang et al. [64]
Bacteria	*Salmonella enterica* serovar Typhimurium	Mouse	Primary, colon	*E. faecium* provides host tolerance to *S.* Typhimurium infection by reducing chemokine secretion and improving host barrier integrity.	Gazzaniga et al. [65]
Bacteria	*Shigella flexneri*	Human	Primary, colon	*S. flexneri* adheres to crypt-like structures and its invasion of the epithelium is enhanced by peristalsis-like mechanical forces.	Grassart et al. [31]
Bacteria	Various (human stool)	Human	Caco-2 cells and primary, ileum	Co-culture of aerobic and anaerobic microbes isolated from human stool consisting of over 200 operational taxonomic units in physiologically relevant ratios.	Jalili-Firoozinezhad et al. [21]
Bacteria	Commensal consortium and enteroinvasive *Escherichia coli* (EIEC)	Human	Caco-2 cells	Co-culture of a defined microbial probiotic community with EIEC improved host epithelial barrier integrity compared to EIEC infection alone. In addition, peristalsis-like forces reduce *E. coli* overgrowth.	Kim et al. [59]
Bacteria	*Lactobacillus rhamnosus* GG	Human	Caco-2 cells	Co-culture with LGG improves epithelial barrier integrity compared to Caco-2 cell monoculture.	Kim et al. [19]
Bacteria	Enterotoxigenic *Bacteroides fragilis and Lactobacillus rhamnosus GG*	Human	Caco-2 cells	LGG limits ETBF infection by directly aggregating with ETBF and interfering with ETBF epithelial attachment.	Lee et al. [66]
Bacteria	*Campylobacter jejuni*	Human	Caco-2 cells	Metabolomics demonstrated alterations in host glycolysis, gluconeogenesis, and amino acid metabolism during *C. jejuni* infection. These changes were not observed during Transwell infections.	Mortensen et al. [49]
Bacteria	*Fusobacterium nucleatum* and fecal microbiota	Human	HCT116 cells	*F. nucleatum* caused transcriptional changes in genes related to cytokine production, proliferation, and epithelial-to-mesenchymal transition. When cultured with a diverse microbiota, *F. nucleatum* infection also decreased the abundance of several anti-inflammatory taxa.	Penarete-Acosta et al. [67]
Bacteria	Enterohemorrhagic *Escherichia coli* (EHEC)	Human	Primary, colon	Four human microbiota metabolites, but not their murine counterparts, increased EHEC infection through upregulation of bacterial motility genes.	Tovaglieri et al. [9]
Bacteria	*Shigella flexneri*	Human	Caco-2 cells	Human enteric α-defensin 5 (HD5) promotes *S. flexneri* infection by activating the P2Y11 receptor and triggering filopodial extensions through the cAMP-PKA pathway.	Xu et al. [68]
Bacteria	*Bifidobacterium breve and Escherichia coli*	Human	Caco-2 cells	*B. breve* colonization prior to *E. coli* infection provided epithelial protection.	Yuan et al. [69]
Bacteria	*Lactobacillus rhamnosus* and *Escherichia coli*	Human	Caco-2 cells	Extended-spectrum resistant *E. coli* induced barrier dysfunction and inflammation, which were mitigated with amikacin treatment, but not with the probiotic *L. rhamnosus.*	Zhao et al. [70]
*Bacterial Enterotoxin	*Escherichia coli* heat-stable enterotoxin A	Human	Primary, jejunal	Fluid flow and stretch forces increased cGMP secretion induced by *E. coli* heat-stable enterotoxin A (ST) in GoC, but not in static culture.	Sunuwar et al. [63]
Virus	Coxsackievirus B1	Human	Caco-2 cells	Coxsackievirus infection displayed fewer cytopathic effects and reduced viral titer when infected from the basolateral compared to apical channels.	Villenave et al. [32]
Virus	*Escherichia coli* phage øPNJ-6 and enterotoxigenic *E. coli* (ETEC)	Human	HT-29 cells	*E. coli* phage, øPNJ-6, bound to MUC2 fucose residues, increased host mucus production, and reduced ETEC infection.	Wu et al. [71]
Virus	Coronavirus	Human	Primary, duodenal	Elevated ACE-2 expression in the presence of flow and peristalsis-like conditions compared to static culture facilitates anti-viral drug screening against the NL63 coronavirus.	Bein et al. [61]
Fungi	*Candida albicans*	Human	Caco-2 cells	Bacteria-activated Vδ2+T-cells inhibit *Candida albicans* outgrowth through an IL-22-dependent mechanism.	Mathew et al. [72]
Bacteria and Fungi	*Lactobacillus rhamnosus* and *Candida albicans*	Human	Caco-2 cells	Pre-colonization by *L. rhamnosus* inhibited subsequent overgrowth by *C. albicans.*	Maurer et al. [73]
Parasite	*Cryptosporidium hominis*	Human	HCT-8 cells	*C. hominis* can be maintained for up to 10 days, supporting completion of its asexual lifecycle with limited sexual reproduction and fertilization.	Gunasekera et al. [62]
Bacteria and Parasite	*Entamoeba histolytica* and *Shigella flexneri*	Human	Caco-2 cells	Peristalsis-like forces increase *E. histolytica* invasion speed and enhance *S. flexneri* colonization by accelerating virulence gene expression.	Boquet-Pujadas et al. [60]

**Table 2:** Infection modeling using ALI.

Pathogen		Cell type		Major findings	Ref
Domain/kingdom	Species	Host species	Source		
Bacteria	*Escherichia coli*	Human	Primary, colon	ALI mucus layer impedes *E. coli* access to the epithelium	Wang et al. [74]
Bacteria	*Clostridioides difficile*	Human	Primary, transverse colon	Mucus promotes *C. difficile* growth, alters bacterial metabolism towards mucus-derived nutrients, and enhances biofilm formation.	Furtado et al. [75]
Bacteria	*Lactobacillus rhamnosus GG* and *Anaerobutyricum hallii*	Human	Primary, transverse colon	ALI cultures support co-culture with anaerobic bacteria *L. rhamnosus GG* and A. hallii while maintaining high cell viability of human intestinal epithelial cells.	Kim et al. [22]
Bacteria	*Escherichia coli*	Human	Primary, sigmoid colon	ALI monolayers grown from ulcerative colitis patient samples form less robust monolayers than those from healthy donors and are more susceptible to pathobiont *E. coli* p19A infection.	Gilliland et al. [48]
Bacteria	*Citrobacter rodentium*	Mouse	Primary, distal colon	Class I Serine protease autotransporter of Enterobacteriaceae (SPATE) EspC from the mouse pathogen *C. rodentium* degrades mucus isolated from mouse ALI but not bovine submaxillary mucus.	Chen et al. [51]
Parasite	*Cryptosporidium parvum*	Mouse	Primary, ileum	ALI cultures support the full life cycle of *C. parvum* thereby identifying a novel reproductive stage, late-stage macrogamonts. Long-term culturing also allowed for the formation of transgenic parasite strains.	Wilke et al. [17]
Parasite	*Trichuris muris* L1 larvae	Mouse	Primary, caecal	*T. muris* L1 larvae degrade intestinal mucus to invade and tunnel through IECs, forming syncytial tunnels.	Duque-Correa et al. [76]
Parasite	*Trichostrongylus colubriformis*	Human	HT-29 cells	*T. colubriformis* displayed tunneling-like movements, induced host Il-33 release, and caused epithelial necrosis during infection.	Andronicos et al. [77]
Virus	Murine astrovirus	Mouse	Primary, small intestine	Murine astroviruses target goblet cells and enterocytes and induce IFN- λ.	Ingle et al. [78]
Virus	Swine enteric coronavirus	Porcine	Ileum	PEDV and TGEV demonstrate reduced infection in ALI monolayer compared to submerged monolayers due to the antiviral effects of sialylated Muc2.	Yang et al. [53]
Virus	SARS-CoV-2	Human	Human pluripotent stem cells	Reduced SARS-CoV-2 infection in fetal-like compared to adult-like ALI monolayers.	Kwon et al. [29]
Virus	Murine norovirus	Mouse	Primary, ileum	Persistent and non-persistent murine noroviruses target tuft cells during infection. Short-lived tuft cells are also required for persistent murine norovirus infections.	Strine et al. [79]
Virus and parasite	*Cryptosporidium parvum, Cryptosporidium hominis* and rotavirus	Human	Primary, ileum	Human ALI cultures support *C. hominis* growth through all life cycle stages, including oocyst development. Both *C. hominis* and *C. parvum* cause type *I* and *III* interferon responses that impair concurrent rotavirus infections.	Greigert et al. [80]

### Bacteria – *C. jejuni*, *C. difficile*, *C. rodentium*, and *S. flexneri*

NGIMs have emerged as powerful *in vitro* platforms for studying fastidious bacterial pathogens. For example, *C. jejuni* is challenging to investigate using mice, due to the robust colonization resistance of their microbiota. GoC systems were used to show that *C. jejuni* disrupts the tight junctions and actin brush border of Caco-2 cells, causing significant cellular damage [49,81]. Similarly, *C. difficile,* an anaerobic pathogen*,* is difficult to study due to its oxygen sensitivity and poor infectivity in mice [82] (Fig 3B). In ALI cultures, exposure to mucus promotes *C. difficile* persistence by shifting their metabolism towards mucus-derived nutrients, while also stimulating biofilm formation and altering the viscoelastic properties of the mucus [75]. Recently, a GoC model involving stable co-colonization of *C. difficile* with the anaerobe *B. fragilis*, revealed that vancomycin treatment led to *C. difficile* closely clustering with vancomycin-resistant *B. fragilis* in a dense biofilm [64]*.* Notably, some *C. difficile* invaded IECs, becoming intracellular, illustrating a potential mechanism by which this pathogen evades antibiotics and potentially leads to infection recurrence [64] (Fig 3B). Thus, ALI and GoC culturing provide a platform to model infections by fastidious pathogens, allowing for a more thorough investigation of virulence mechanisms that could inform therapeutic development.

Advanced *in vitro* systems have also uncovered new virulence mechanisms used by bacterial pathogens, despite being well-studied using traditional models. For example, exposing *C. rodentium* to ALI-derived mucus identified a previously unrecognized mucolytic function for the serine protease EspC [51]. GoC studies also revealed that *S. flexneri* microcolonies start from a single bacterium and subsequently invade IECs via direct intracellular basal translocation, not through a paracellular route as previously thought [31]. Another GoC study demonstrated that *S. flexneri* exploits human antimicrobial peptide alpha defensin 5 (HD5)-induced filopodial extension of IECs for invasion [68]. As such, ALI and GoC platforms can also provide novel insights into well-studied bacterial species.

### Fungi *– C. albicans*

Although fungal species are increasingly recognized for their contributions to chronic infections and intestinal disease, their pathogenic mechanisms remain poorly understood, largely due to limitations in *in vivo* models [72,83,84]. *Candida albicans*, a gut commensal and opportunistic pathobiont, cannot colonize mice without antibiotic pre-treatment, resulting in a model of limited relevance, as the microbiota modulate *C. albicans* virulence [72]. As observed in GoC models, *C. albicans* undergoes yeast-to-hyphal transition and infects IECs; however, pre-colonization with *Lactobacillus rhamnosus* suppresses this process, reducing fungal burden and translocation, as well as tissue damage [72,73,85]. Since *C. albicans* pathogenicity heavily relies on interactions with commensal microbes, ALI or GoC models with anaerobic compartments could help define the factors governing *C. albicans* transition from commensal to pathobiont, and how the gut microbiota confers host protection. Such studies would be helpful in determining how *C. albicans* colonizes immunocompromised patients with gut dysbiosis, as they often suffer life-threatening infections [86].

### Parasites *– C. parvum, E. histolytica, T. muris*

Parasite infections have also been extensively investigated in NGIMs. Following infection of mouse-derived ALI monolayers, *Cryptosporidium parvum* underwent full development, expanding 100-fold and generating viable oocysts. Notably, these conditions revealed a previously unidentified *C. parvum* life stage, termed “late-stage macrogamonts” that had not been observed in conventional cell cultures [17] (Fig 3C). It also allowed for *in vitro* genetic crossing of *C. parvum*, thereby opening avenues for forward genetics without relying on animal hosts [17]. ALI cultures were also found to support the growth and development of the human-specific *Cryptosporidium hominis* [80]. Notably, GoC systems also permit long-term culturing of both *C. parvum* and *C. hominis*, ranging from 10 days to 4 weeks, allowing multiple stages of parasite development [62,87,88].

Helminths, another important class of gastrointestinal pathogens, present unique challenges for *in vitro* modeling due to their large size and complex lifecycles involving tissue invasion and molting. Attempts to model *Trichuris muris* infection in conventional 3D organoids did not support IEC invasion and colonization [89]. In contrast, organoid-derived Transwell cultures that closely resemble ALI cultures, captured *T. muris* L1 larvae degrading the overlying mucus layer, invading IECs, and forming syncytial tunnels characteristic of infection i*n vivo* [76]. The capacity for ALI cultures to model helminth-epithelial interactions has also been demonstrated using *Trichostrongylus colubriformis,* in which L3 larvae displayed tunneling-like movements in HT-29 cells and induced epithelial necrosis [77]. More recently, live imaging of *T. muris* infection in microfluidic devices showed that L1 larvae tunnel through caecal epithelial cells toward the base of crypts, causing localized IEC death [90]. Although current application of NGIMs towards helminth infections remains limited, these systems offer considerable potential to model the complex lifecycle and infection strategies of parasites.

### Viruses *–* coronavirus, rotavirus, murine astrovirus, and norovirus

Modeling human enteric virus infections in animal models can be challenging as there are important species differences in the receptors expressed by their IECs [32]. ALI and GoC models can overcome this limitation by enabling the use of IECs from relevant hosts. For example, by using porcine ALI monolayers, researchers determined that susceptibility to transmissible gastroenteritis virus (TGEV) and porcine epidemic diarrhea virus (PEDV) was reduced, as compared to submerged monolayer cultures, due to the antiviral effects of sialylated mucus [53]. Similarly, human iPSC-derived ALI monolayers resembling fetal-like intestinal tissue were found to express less ACE2, the receptor for SARS-CoV-2, than adult-like tissues, resulting in lower infection rates [29]. This observation is consistent with the milder COVID-19 severity seen in pediatric populations [91]. In contrast, adult organoids grown in GoC show increased ACE2 as compared to 3D organoids, making GoC systems superior for antiviral drug screening [61] (Fig 3D). Despite their clinical importance as enteric pathogens, relatively few studies have investigated norovirus and rotavirus pathogenesis using ALI or GoC systems. Although murine norovirus infections have not been successfully modeled in murine enteroids, mouse-derived intestinal ALI cultures have supported infection, revealing tuft cell tropism and a mechanism by which tuft cells serve as a reservoir for persistent norovirus infection [79]. Human intestinal ALI cultures have also been used to model *C. parvum-*rotavirus coinfection, revealing that parasite-induced type III interferon responses reduced subsequent rotavirus infection [80]. Expanding the application of ALI and GoC systems to the study of human norovirus and rotavirus remains an important area for future investigation. These findings suggest that ALI and GoC systems can model important host-specific aspects of physiology when investigating viral host specificity, infection dynamics, and anti-viral therapeutic discovery.

### Pathogen-commensal interactions

As noted above, the addition of gut microbiota to these models would be beneficial in terms of defining their impact on pathogen virulence. Several GoC studies have confirmed that individual commensal strains can suppress pathogenic *E. coli* infections [69,92]. For example, the common probiotic strain *Lactobacillus rhamnosus* GG (LGG) was shown to proliferate along crypt- and villi-like structures in GoC models and co-aggregated with enterotoxigenic *Bacteroides fragilis* (ETBF), effectively impairing ETBF pathogenicity [66]. Moving to complex microbiota consortia, the metabolites from human, but not murine, gut microbiota were shown to influence enterohemorrhagic *E. coli* (EHEC) pathogenesis in human GoC models, by inducing expression of EHEC’s chemotaxis and motility genes, resulting in increased damage to IECs [9]. In contrast, *Enterococcus* species within a complex microbiota were found to promote host tolerance against *S. Typhimurium* infection by improving tight junction integrity, while a diverse commensal microbiota substantially dampened the inflammatory response induced by *Fusobacterium nucleatum* infection in GoC systems [65,67]. Despite these observations, the microbial communities used in NGIMs remain substantially less complex than the native intestinal microbiome, and their long-term stability within NGIMs remains limited. Nevertheless, these GoC-based studies demonstrate that both individual commensal strains and defined microbial communities can modulate pathogen and host responses, allowing for the exploration of microbiota-based therapeutic approaches against enteric pathogens.

## ALI vs GoC: Which model is right for me?

ALI cultures and GoC systems both provide more physiologically relevant human-based platforms to study enteric pathogens than traditional cell culture and animal models. We next discuss key factors that potential users should consider when choosing between NGIMs for enteric infection modeling.

### Modeling physiological factors

While both ALI and GoC promote IEC differentiation, they do so through distinct pathways. ALI cultures rely on increased oxygen tension driving a metabolic shift in IECs from glycolysis to oxidative phosphorylation, whereas GoC devices use luminal flow and stretch to promote IEC maturation [16,20,93]. Future studies should directly compare IEC differentiation in ALI and GoC systems to better guide model selection when specific IEC subtypes are required. While both systems produce a secreted mucus layer, ALI mucus is more readily accessible as compared to GoC, which typically requires chip disassembly [50,51]. This ease of access with ALI facilitates *in situ* rheology analysis of mucus viscosity during infections [94]. Additionally, ALI conditions may promote a thicker and more protective mucus layer. For example, Caco-2 cells grown on microfluidic devices under ALI conditions displayed higher MUC2 expression, increased cell viability, and greater cell layer thickness as compared to microfluidic, liquid-liquid interface conditions [95]. Although direct mucus comparisons between ‘pure’ ALI conditions and GoC systems have yet to be performed, these findings support ALI cultures as a particularly attractive platform when mucus is a point of interest in infection studies. In contrast, GoC systems better model intestinal peristalsis and luminal flow, both key contributors to pathogen clearance [19]. Other influencing factors such as anaerobic co-culturing capabilities, crypt or villi structures, and incorporation of immune and stromal cells are possible in both systems (Fig 2). Beyond the physical platform, the cellular source used to generate NGIMs is another important consideration. Cell lines are accessible and largely reproducible but lack the cellular diversity of primary intestinal epithelium. iPSCs offer considerable scalability and typically display a less mature, fetal-like phenotype, which may be advantageous for studying early-life infections and how they may induce persistent epithelial remodeling, but offer limited relevance to the study of adult physiology [96,97]. In contrast, tissue-derived intestinal stem cells better preserve the regional characteristics of their tissue origin, including disease-associated features [18,48].

### Ease of access

Beyond biological factors, accessibility and scalability also differ between ALI and GoC systems. GoC systems require greater technical expertise, optimization, and financial investment. Beyond the high cost of microfluidic chips, GoC systems also require dedicated infrastructure including pumps and flow controllers that reduce throughput and raise set-up costs [98]. In contrast, ALI cultures provide a cost-effective alternative by utilizing standard lab equipment (i.e., Transwells), thereby offering greater scalability and high-throughput potential. Thus, ALI cultures serve as the more accessible entry point for laboratories seeking advanced infection modeling methods, without the financial and technical barriers of GoC systems. To address limited access to primary cells from human biopsies or animal tissues, labs establishing NGIMs can start by using well-established IEC lines, like Caco-2 cells, which already demonstrate greater physiological relevance when grown in NGIMs as compared to their growth as conventional monolayer cultures [99].

## Limitations

Despite their improved physiological relevance, NGIMs are inherently reductionist and cannot fully recapitulate the systemic nature of *in vivo* infections and host responses. Most notably, current systems lack a fully functional and integrated immune component and cannot capture processes such as immune cell priming and their subsequent recruitment during infection. NGIMs also lack interactions with distal organs and may thereby fail to reproduce systemic responses that may influence pathology. These limitations should be considered when interpreting NGIM infection studies, as reduced immune defenses and nutrient-rich culturing conditions may create a permissive environment for microbial growth that does not recapitulate *in vivo* conditions. Moreover, while NGIMs can incorporate human commensal microbes, they do not yet support a stable, complex microbiome as seen *in vivo*. Conversely, although live rodent models maintain a complex intestinal microbiota, their composition and function can substantially differ from the gut microbiota of humans. Thus, depending on the biological question being asked, NGIMs should currently be viewed as complementary to, rather than a complete replacement for *in vivo* models.

## Model advancements on the horizon

ALI and GoC systems have undoubtedly improved our abilities to model the native human intestine, yielding new insights into pathogen-host interactions. Here we discuss how these models can be improved both to increase their relevance through incorporation of supporting cell types and specialized technical advancements, as well as improving accessibility and standardization.

### Beyond the epithelium – Immune, stromal, and ENS inclusion

Beyond IECs, immune and stromal cells are also key to an effective host defense against enteric pathogens [100]. Researchers have recently begun integrating stromal and immune cell compartments into ALI and GoC systems. For example, human duodenal ALI cultures with autologous stromal and tissue-resident immune cells (B, T, NK, and myeloid cells) were found to recapitulate the *in vivo* immune-mediated enteropathy of celiac disease, while GoC systems have incorporated IEC-stromal co-cultures as proof of concept [101–103]. Incorporating tissue-resident immune and stromal compartments into ALI and GoC platforms for infection modeling could help identify novel mechanisms by which pathogens evade immunity. Beyond simple co-culture, it will be critical for these cell types to recapitulate their 3D spatial organization and dynamic interactions observed *in vivo,* rather than functioning as isolated compartments. These complex co-cultures will require the optimization of culturing media composition to support all the various specialized cells. Coupling these advances with increasingly complex bacterial and fungal communities could enable better modeling of how the intestinal microbiome shapes both immune function and susceptibility to enteric infection. Moreover, these additions could help elucidate the potential role of pathobionts in triggering or exacerbating chronic immune-mediated inflammation as seen in IBD and other diseases [83]. The enteric nervous system (ENS) is another component of the GI tract that influences enteric infection kinetics. For example, the ENS can modulate mucosal barriers during infection by promoting antimicrobial peptide secretion from goblet cells [104]. The ENS can also be exploited by various pathogens; for example, *C. difficile* toxins disrupt the ENS, thereby reducing GI mobility and facilitating pathogen colonization [105,106]. Thus far, only one GoC study has co-cultured iPSC-derived enteric neurons with microbes, specifically *L. reuteri*, thus demonstrating the feasibility of such integration [107]. Incorporating the ENS into infection models may clarify how infections perturb enteric neural function and how enteric pathogens may promote the development of disorders such as PI-IBS [108]. Fully integrating the ENS, stromal and immune compartments into ALI and GoC systems will enable physiologically relevant multicompartmental crosstalk, better reflecting the complexity of the human GI tract beyond the mucosal surface.

### Specialized technical advancements

Advancements in NGIMs should also prioritize expanding their technical capabilities. Mouse models have long benefited from well-established genetic editing technologies. Recently, an iPSC-derived intestinal reporter line was generated and maintained under ALI conditions, proving the feasibility of genetic editing in these advanced models [29]. In GoC, integration of real-time sensors for transepithelial electrical resistance (TEER), pH, and oxygen gradient measurements has recently been established and will provide improved temporal resolution of infection dynamics [109]. Incorporation of artificial intelligence (AI) and machine learning technologies could also increase throughput and improve imaging analysis [110]. Because enteric pathogens and gut microbial dysbiosis contribute to systemic pathology in other organs including the liver, kidneys, and heart, extending ALI and GoC into multi-organ systems is also vital [111]. Multi-organ microfluidic systems have already been developed and were used to successfully model Shiga toxin-producing *E. coli* (STEC)-induced hemolytic-uremic syndrome [112]. Future efforts should try to address how enteric pathogens and gut dysbiosis may influence disease progression beyond the GI tract.

### A need for access and standardization

Additional advancements in the field should focus on improving the accessibility and standardization of ALI and GoC. A major impediment to the widespread application of GoC is the cost of microfluidic chips and their supporting hardware. Fortunately, recent advances have led to cheaper microfluidic chip fabrication via 3D bioprinting, the elimination of expensive hardware, as well as high-throughput formats [98,113,114].

Standardization is also an important aspect of NGIMs that should be prioritized as these systems gain more popularity. Differences in cell source, media composition, extracellular matrix (ECM) substrate, and culturing protocols can all confound results when trying to draw comparisons between studies. Interestingly, patient-derived submerged monolayers and 3D organoids show only modest donor-donor variation in transcriptomic responses to infection, whereas factors such as passage number, media composition, and ECM substrate (Matrigel or collagen) cause greater differences [115,116]. This underscores the need for caution when comparing results across labs and systems. Future studies should prioritize establishing standardized protocols, especially regarding ECM substrates and media, to improve inter-lab reproducibility. Such efforts have started for 3D organoids through the NIH Standard Organoid Modeling Center and other consortiums and should be extended for NGIMs [117]. Moreover, multiple labs utilizing the same large organoid biobanks may reduce inter-laboratory variations, though the natural variations seen between donors may also model patient-specific responses. To capitalize on these differences, biobanks should also incorporate molecular characterization of tissue donors. Linking a donor’s clinical data with multi-omic profiles and phenotypes observed in NGIMs could help identify patient-specific determinants of infection susceptibility and treatment response. Advancing the physiological properties of NGIMs, expanding their toolkit, and improving both accessibility and standardization should improve infection modeling across the field by helping foster widespread integration of these techniques in concert with increasingly reliable and reproducible results.

## Conclusions

ALI and GoC systems display many advantages over conventional cell culture by more accurately recapitulating the human intestinal mucosal surface, including a robust mucus barrier, realistic architecture, and a mature epithelium. These models have improved our understanding of how enteric pathogens penetrate mucus, adhere to the intestinal epithelium, and replicate under defined biomechanical forces such as shear stress and luminal flow. Importantly, both systems enable complex co-culture studies, including co-infections and pathogen-commensal co-culture, which were previously challenging to pursue in traditional *in vitro* models. Notably, NGIMs offer the added benefit of using human cells, providing novel insights into microbe-human specific interactions. Recent advancements have begun incorporating other cellular compartments found below the mucosal surface, to further address understudied aspects of host-pathogen dynamics. While this review focuses on ALI and GoC systems, we acknowledge that other advanced GI models, including scaffold- or hydrogel-based systems, also offer improved recapitulation of intestinal physiology as compared to traditional models; however, their application towards infection modeling is thus far very limited [88,118].

Both ALI and GoC systems show great potential for precision medicine. Under ALI conditions, monolayers grown from the colonic biopsies of patients with ulcerative colitis were found to mimic disease phenotypes *in vitro,* including enhanced susceptibility to *E. coli* pathobiont infection [48]. Long-term co-culture of patient-derived intestinal epithelium with an autologous microbiome in NGIMs could inform the selection of antimicrobial regimens that minimize microbiome disruption, thereby reducing the risk of *C. difficile* and other post-antibiotic infections. As such, ALI and GoC systems can serve as individualized models for various personalized medicine applications.

Given the urgent need for alternatives to conventional antibiotics, NGIMs could prove to be powerful platforms for the targeted evaluation of next-generation antimicrobials. Their capacity for long-term culturing should facilitate the evaluation of probiotics, fecal microbiota transplantation (FMT), and other microbiota-modulating interventions, as well as their impact on an enteric pathogen’s ability to colonize and cause disease, in both preventative and therapeutic contexts [119]. The presence of a physiological mucus layer, vital for bacteriophage adhesion, also makes these models well suited for phage therapy studies [71,120]. Likewise, the mucus in these systems increases their relevance for evaluating antimicrobial peptides, many of which have enhanced anti-bacterial and anti-biofilm activity in the presence of mucus [121]. Other antimicrobial therapies, including anti-adhesin and host-directed therapies, would also benefit from screening in ALI and GoC platforms as they provide greater physiological fidelity than traditional models (Fig 4). NGIMs are therefore extremely powerful and versatile tools for infection research, bridging the gap between conventional methods and the complexities of the human gut to provide novel insights into the virulence as well as weaknesses of enteric pathogens that pose a global threat to human health.

**Fig 4:**
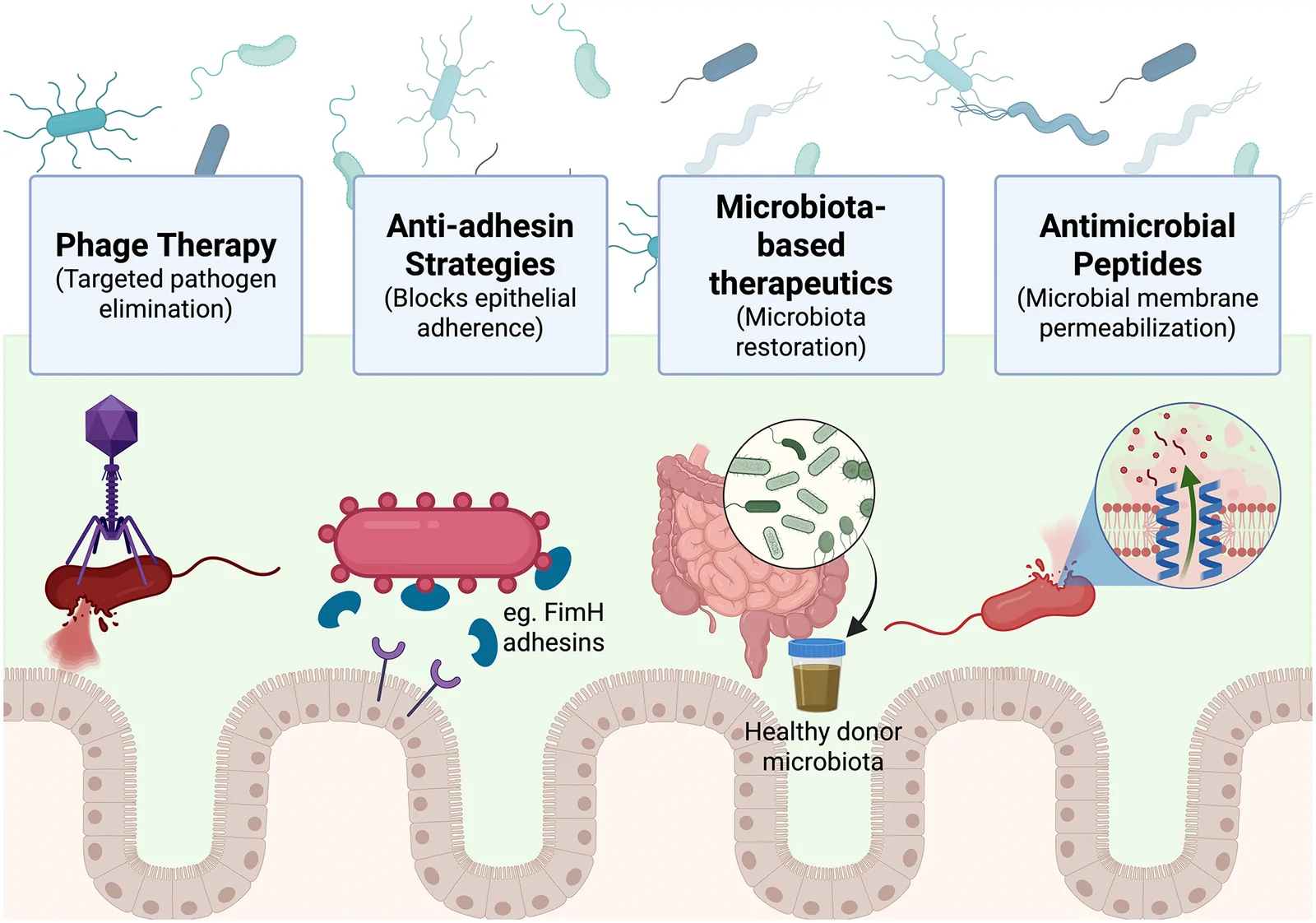
ALI and GoC systems for evaluating novel therapeutics against enteric infections. Schematic of NGIMs for preclinical evaluation of various next-generation antimicrobial therapies. This includes phage therapy, which selectively lyses or impairs the function of pathogenic bacteria; anti-adhesin therapies, such as FimH adhesins, which prevent pathogen attachment to the epithelium; microbiota-based therapies, such as fecal microbiota transplantation (FMT) and probiotics, which restore microbiota composition and provide colonization resistance against pathogens; and antimicrobial peptides, which disrupt bacterial membranes, suppressing the growth or killing specific pathogens. Created in BioRender. Vallance, B. (2026) https://BioRender.com/me5bsrb.

## References

[1] Kyu HH, Vongpradith A, Dominguez R-MV, Ma J, Albertson SB, Novotney A. Global, regional, and national age-sex-specific burden of diarrhoeal diseases, their risk factors, and aetiologies, 1990–2021, for 204 countries and territories: A systematic analysis for the Global Burden of Disease Study 2021. Lancet Infect Dis. 2025;25:519–36. doi: 10.1016/S1473-3099(24)00691-1 PMC12018300 39708822

[2] Li T, Qiang N, Bao Y, Li Y, Zhao S, Chong KC, et al. Global burden of enteric infections related foodborne diseases, 1990-2021: Findings from the Global Burden of Disease Study 2021. Sci One Health. 2024;3:100075. doi: 10.1016/j.soh.2024.100075 39282625 PMC11402448

[3] Arastehfar A, Gabaldón T, Garcia-Rubio R, Jenks JD, Hoenigl M, Salzer HJF, et al. Drug-resistant fungi: An emerging challenge threatening our limited antifungal armamentarium. Antibiotics (Basel). 2020;9(12):877. doi: 10.3390/antibiotics9120877 33302565 PMC7764418

[4] Wallace MJ, Fishbein SRS, Dantas G. Antimicrobial resistance in enteric bacteria: Current state and next-generation solutions. Gut Microbes. 2020;12(1):1799654. doi: 10.1080/19490976.2020.1799654 32772817 PMC7524338

[5] Axelrad JE, Cadwell KH, Colombel J-F, Shah SC. The role of gastrointestinal pathogens in inflammatory bowel disease: A systematic review. Ther Adv Gastroenterol. 2021;14:17562848211004493. doi: 10.1177/17562848211004493 PMC8020742 33868457

[6] Sadeghi A, Biglari M, Nasseri Moghaddam S. Post-infectious irritable bowel syndrome: A narrative review. Middle East J Dig Dis. 2019;11:69–75. doi: 10.15171/mejdd.2019.130 PMC6663289 31380002

[7] Lesuffleur T, Porchet N, Aubert JP, Swallow D, Gum JR, Kim YS, et al. Differential expression of the human mucin genes MUC1 to MUC5 in relation to growth and differentiation of different mucus-secreting HT-29 cell subpopulations. J Cell Sci. 1993;106 (Pt 3):771–83. doi: 10.1242/jcs.106.3.771 8308060

[8] van Klinken BJ, Oussoren E, Weenink JJ, Strous GJ, Büller HA, Dekker J, et al. The human intestinal cell lines Caco-2 and LS174T as models to study cell-type specific mucin expression. Glycoconj J. 1996;13(5):757–68. doi: 10.1007/BF00702340 8910003

[9] Tovaglieri A, Sontheimer-Phelps A, Geirnaert A, Prantil-Baun R, Camacho DM, Chou DB, et al. Species-specific enhancement of enterohemorrhagic *E. coli* pathogenesis mediated by microbiome metabolites. Microbiome. 2019;7(1):43. doi: 10.1186/s40168-019-0650-5 30890187 PMC6425591

[10] Taube S, Kolawole AO, Höhne M, Wilkinson JE, Handley SA, Perry JW, et al. A mouse model for human norovirus. mBio. 2013;4(4):e00450-13. doi: 10.1128/mBio.00450-13 23860770 PMC3735125

[11] Fleckenstein JM, Rasko DA. Overcoming enterotoxigenic *Escherichia coli* pathogen diversity: Translational molecular approaches to inform vaccine design. Methods Mol Biol. 2016;1403:363–83. doi: 10.1007/978-1-4939-3387-7_19 27076141 PMC5228307

[12] Franklin CL, Ericsson AC. Microbiota and reproducibility of rodent models. Lab Anim (NY). 2017;46(4):114–22. doi: 10.1038/laban.1222 28328896 PMC5762113

[13] Han JJ. FDA modernization act 2.0 allows for alternatives to animal testing. Artif Organs. 2023;47:449–50. doi: 10.1111/aor.14503 36762462

[14] Han X, Mslati MA, Davies E, Chen Y, Allaire JM, Vallance BA. Creating a more perfect union: Modeling intestinal bacteria-epithelial interactions using organoids. Cell Mol Gastroenterol Hepatol. 2021;12(2):769–82. doi: 10.1016/j.jcmgh.2021.04.010 33895425 PMC8273413

[15] Wang Y, Kim R, Sims CE, Allbritton NL. Building a thick mucus hydrogel layer to improve the physiological relevance of in vitro primary colonic epithelial models. CMGH. 2019;8: 653–5. doi: 10.1016/j.jcmgh.2019.07.009 PMC6889783 31356887

[16] Wang X, Yamamoto Y, Wilson LH, Zhang T, Howitt BE, Farrow MA, et al. Cloning and variation of ground state intestinal stem cells. Nature. 2015;522(7555):173–8. doi: 10.1038/nature14484 26040716 PMC4853906

[17] Wilke G, Funkhouser-Jones LJ, Wang Y, Ravindran S, Wang Q, Beatty WL, et al. A stem-cell-derived platform enables complete cryptosporidium development in vitro and genetic tractability. Cell Host Microbe. 2019;26: 123–34. doi: 10.1016/j.chom.2019.05.007 PMC6617391 31231046

[18] Wang Y, Chiang I-L, Ohara TE, Fujii S, Cheng J, Muegge BD, et al. Long-term culture captures injury-repair cycles of colonic stem cells. Cell. 2019;179(5):1144-1159.e15. doi: 10.1016/j.cell.2019.10.015 31708126 PMC6904908

[19] Kim HJ, Huh D, Hamilton G, Ingber DE. Human gut-on-a-chip inhabited by microbial flora that experiences intestinal peristalsis-like motions and flow. Lab Chip. 2012;12(12):2165–74. doi: 10.1039/c2lc40074j 22434367

[20] Kasendra M, Tovaglieri A, Sontheimer-Phelps A, Jalili-Firoozinezhad S, Bein A, Chalkiadaki A, et al. Development of a primary human Small Intestine-on-a-Chip using biopsy-derived organoids. Sci Rep. 2018;8(1):2871. doi: 10.1038/s41598-018-21201-7 29440725 PMC5811607

[21] Jalili-Firoozinezhad S, Gazzaniga FS, Calamari EL, Camacho DM, Fadel CW, Bein A, et al. A complex human gut microbiome cultured in an anaerobic intestine-on-a-chip. Nat Biomed Eng. 2019;3(7):520–31. doi: 10.1038/s41551-019-0397-0 31086325 PMC6658209

[22] Kim R, Allbritton NL. A microphysiological system with an anaerobic air-liquid interface and functional mucus layer for coculture of intestinal bacteria and primary human colonic epithelium. Adv Mater Interfaces. 2024;11(25):2400093. doi: 10.1002/admi.202400093 39386255 PMC11460523

[23] Huang J, Zhou C, Zhou G, Li H, Ye K. Effect of *Listeria monocytogenes* on intestinal stem cells in the co-culture model of small intestinal organoids. Microb Pathog. 2021;153:104776. doi: 10.1016/j.micpath.2021.104776 33548482

[24] Mileto SJ, Jardé T, Childress KO, Jensen JL, Rogers AP, Kerr G, et al. *Clostridioides difficile* infection damages colonic stem cells via TcdB, impairing epithelial repair and recovery from disease. Proc Natl Acad Sci U S A. 2020;117(14):8064–73. doi: 10.1073/pnas.1915255117 32198200 PMC7149309

[25] Pentecost M, Kumaran J, Ghosh P, Amieva MR. *Listeria monocytogenes* internalin B activates junctional endocytosis to accelerate intestinal invasion. PLoS Pathog. 2010;6(5):e1000900. doi: 10.1371/journal.ppat.1000900 20485518 PMC2869327

[26] Rodrigues E, Pallett MA, Straker LC, Mkandawire TT, Sala K, Collinson L, et al. Cryptosporidium modifies intestinal microvilli through an exported virulence factor. Cell Host Microbe. 2025;33(5):719-730.e5. doi: 10.1016/j.chom.2025.04.001 40300595 PMC7618952

[27] Ferreira B, Barros AS, Leite-Pereira C, Viegas J, das Neves J, Nunes R, et al. Trends in 3D models of inflammatory bowel disease. Biochim Biophys Acta Mol Basis Dis. 2024;1870(3):167042. doi: 10.1016/j.bbadis.2024.167042 38296115

[28] Nguyen TLA, Vieira-Silva S, Liston A, Raes J. How informative is the mouse for human gut microbiota research? Dis Model Mech. 2015;8(1):1–16. doi: 10.1242/dmm.017400 25561744 PMC4283646

[29] Kwon O, Lee H, Jung J, Son YS, Jeon S, Yu WD, et al. Chemically-defined and scalable culture system for intestinal stem cells derived from human intestinal organoids. Nat Commun. 2024;15(1):799. doi: 10.1038/s41467-024-45103-7 38280855 PMC10821882

[30] Ogawa I, Nakai T, Iwao T, Matsunaga T. Air-liquid interface culture combined with differentiation factors reproducing intestinal cell structure formation in vitro. Biol Open. 2025;14(1):bio061612. doi: 10.1242/bio.061612 39832187 PMC11789277

[31] Grassart A, Malardé V, Gobaa S, Sartori-Rupp A, Kerns J, Karalis K, et al. Bioengineered human organ-on-chip reveals intestinal microenvironment and mechanical forces impacting Shigella infection. Cell Host Microbe. 2019;26(3):435-444.e4. doi: 10.1016/j.chom.2019.08.007 31492657

[32] Villenave R, Wales SQ, Hamkins-Indik T, Papafragkou E, Weaver JC, Ferrante TC, et al. Human gut-on-a-chip supports polarized infection of coxsackie B1 virus in vitro. PLoS One. 2017;12(2):e0169412. doi: 10.1371/journal.pone.0169412 28146569 PMC5287454

[33] Papafragkou E, Hewitt J, Park GW, Greening G, Vinjé J. Challenges of culturing human norovirus in three-dimensional organoid intestinal cell culture models. PLoS One. 2013;8(6):e63485. doi: 10.1371/journal.pone.0063485 23755105 PMC3670855

[34] Drummond CG, Bolock AM, Ma C, Luke CJ, Good M, Coyne CB. Enteroviruses infect human enteroids and induce antiviral signaling in a cell lineage-specific manner. Proc Natl Acad Sci U S A. 2017;114(7):1672–7. doi: 10.1073/pnas.1617363114 28137842 PMC5320971

[35] Wang D, Spoelstra WK, Lin L, Akkerman N, Krueger D, Dayton T, et al. Interferon-responsive intestinal BEST4/CA7+ cells are targets of bacterial diarrheal toxins. Cell Stem Cell. 2025;32(4):598-612.e5. doi: 10.1016/j.stem.2025.02.003 40010349

[36] Kim HJ, Ingber DE. Gut-on-a-Chip microenvironment induces human intestinal cells to undergo villus differentiation. Integr Biol (Camb). 2013;5(9):1130–40. doi: 10.1039/c3ib40126j 23817533

[37] Fujii S, Espenschied ST, Anand V, Bettencourt-Silva J, Han Y, Ito G. A high-fidelity in vitro model of the human intestinal epithelium. Biorxiv. 2024. doi: 10.1101/2024.01.01.573838

[38] Moerkens R, Mooiweer J, Smits E, Berg M, Ramírez-Sánchez AD, Modderman R, et al. Gene expression profiling reveals enhanced nutrient and drug metabolism and maturation of hiPSC-derived intestine-on-chip relative to organoids and transwells. Stem Cell Reports. 2025;20(12):102715. doi: 10.1016/j.stemcr.2025.102715 41237783 PMC12744852

[39] Malonga T, Lhuillier E, Marrauld C, Fourmy D, Riant E, Cabau C, et al. Single-cell transcriptomics reveals that air-liquid interface culture promotes goblet cell differentiation and inhibits glycolysis in organoid cell monolayers. Am J Physiol Gastrointest Liver Physiol. 2026;330(5):G513–31. doi: 10.1152/ajpgi.00251.2025 41830459

[40] Wehl I, Michiba K, Walser V, Beck S, Tayfour H, Imaoka T, et al. Characterization of human intestinal organoid-derived monolayers under air-liquid interface culture for pharmacokinetics evaluation. Drug Metab Dispos. 2026;54(6):100308. doi: 10.1016/j.dmd.2026.100308 42190309

[41] Johansson MEV, Gustafsson JK, Holmén-Larsson J, Jabbar KS, Xia L, Xu H, et al. Bacteria penetrate the normally impenetrable inner colon mucus layer in both murine colitis models and patients with ulcerative colitis. Gut. 2014;63(2):281–91. doi: 10.1136/gutjnl-2012-303207 23426893 PMC3740207

[42] Sicard J-F, Le Bihan G, Vogeleer P, Jacques M, Harel J. Interactions of intestinal bacteria with components of the intestinal mucus. Front Cell Infect Microbiol. 2017;7:387. doi: 10.3389/fcimb.2017.00387 28929087 PMC5591952

[43] Trillo-Muyo S, Dolan B, Svensson F, Vickers TJ, Arike L, Garcia-Bonete MJ. Degradation of the intestinal mucus layer by the ETEC protease EatA is species specific determined by the structure of the MUC2 mucin. bioRxiv. 2025:2025.03.16.643548. doi: 10.1101/2025.03.16.643548

[44] Gustafsson JK, Ermund A, Johansson MEV, Schütte A, Hansson GC, Sjövall H. An ex vivo method for studying mucus formation, properties, and thickness in human colonic biopsies and mouse small and large intestinal explants. Am J Physiol Gastrointest Liver Physiol. 2012;302(4):G430-8. doi: 10.1152/ajpgi.00405.2011 22159279 PMC4073982

[45] Johansson MEV, Larsson JMH, Hansson GC. The two mucus layers of colon are organized by the MUC2 mucin, whereas the outer layer is a legislator of host-microbial interactions. Proc Natl Acad Sci U S A. 2011;108 Suppl 1(Suppl 1):4659–65. doi: 10.1073/pnas.1006451107 20615996 PMC3063600

[46] Sardelli L, Pacheco DP, Ziccarelli A, Tunesi M, Caspani O, Fusari A, et al. Towards bioinspired in vitro models of intestinal mucus. RSC Adv. 2019;9(28):15887–99. doi: 10.1039/c9ra02368b 35521409 PMC9064393

[47] Floor E, Su J, Chatterjee M, Kuipers ES, IJssennagger N, Heidari F, et al. Development of a Caco-2-based intestinal mucosal model to study intestinal barrier properties and bacteria-mucus interactions. Gut Microbes. 2025;17(1):2434685. doi: 10.1080/19490976.2024.2434685 39714032 PMC11702969

[48] Gilliland A, Chen Y, Ng I, Han X, Sham HP, Kalisky I, et al. Biopsy-derived colonoid air-liquid interface monolayers reveal persistent mucosal defects in ulcerative colitis patients. Inflamm Bowel Dis. 2026;32(3):526–41. doi: 10.1093/ibd/izaf318 41589595

[49] Mortensen NP, Mercier KA, McRitchie S, Cavallo TB, Pathmasiri W, Stewart D, et al. Microfluidics meets metabolomics to reveal the impact of *Campylobacter jejuni* infection on biochemical pathways. Biomed Microdevices. 2016;18(3):51. doi: 10.1007/s10544-016-0076-9 27231016 PMC4939818

[50] Sontheimer-Phelps A, Chou DB, Tovaglieri A, Ferrante TC, Duckworth T, Fadel C, et al. Human colon-on-a-chip enables continuous in vitro analysis of colon mucus layer accumulation and physiology. Cell Mol Gastroenterol Hepatol. 2020;9: 507–26. doi: 10.1016/j.jcmgh.2019.11.008 PMC7036549 31778828

[51] Chen Y, Gilliland A, Liang Q, Han X, Yang H, Chan J, et al. Defining enteric bacterial pathogenesis using organoids: *Citrobacter rodentium* uses EspC, an atypical mucinolytic protease, to penetrate mouse colonic mucus. Gut Microbes. 2025;17(1):2494717. doi: 10.1080/19490976.2025.2494717 40323239 PMC12054374

[52] Howard RL, Markovetz M, Wang Y, Ehre C, Sheikh SZ, Allbritton NL, et al. Biochemical and rheological analysis of human colonic culture mucus reveals similarity to gut mucus. Biophys J. 2021;120(23):5384–94. doi: 10.1016/j.bpj.2021.10.024 34695384 PMC8715165

[53] Yang N, Li Y, Cai Y, Liu Y, Zhang Y, Fu Y, et al. A mucus layer derived from porcine intestinal organoid air-liquid interface monolayer attenuates swine enteric coronavirus infection by antiviral activity of Muc2. BMC Biol. 2024;22(1):297. doi: 10.1186/s12915-024-02094-7 39716180 PMC11668090

[54] Abdollahi S, Zarin B, Vatani M, Vajhadin F, Hassani M, Jalali P, et al. Biomimetic culture substrates for modelling homeostatic intestinal epithelium in vitro. Nat Commun. 2025;16(1):4120. doi: 10.1038/s41467-025-59459-x 40316543 PMC12048609

[55] Chi M, Yi B, Oh S, Park D-J, Sung JH, Park S. A microfluidic cell culture device (μFCCD) to culture epithelial cells with physiological and morphological properties that mimic those of the human intestine. Biomed Microdevices. 2015;17(3):9966. doi: 10.1007/s10544-015-9966-5 26002774

[56] Lee SY, Byun HJ, Choi H, Won J-I, Han J, Park S, et al. Development of a pumpless microfluidic system to study the interaction between gut microbes and intestinal epithelial cells. Biotechnol Bioproc E. 2022;27(2):221–33. doi: 10.1007/s12257-021-0268-x

[57] Liu J, Lu R, Zheng X, Hou W, Wu X, Zhao H, et al. Establishment of a gut-on-a-chip device with controllable oxygen gradients to study the contribution of *Bifidobacterium bifidum* to inflammatory bowel disease. Biomater Sci. 2023;11(7):2504–17. doi: 10.1039/d2bm01490d 36779280

[58] Zhang J, Huang Y-J, Yoon JY, Kemmitt J, Wright C, Schneider K, et al. Primary human colonic mucosal barrier crosstalk with super oxygen-sensitive *Faecalibacterium prausnitzii* in continuous culture. Med. 2021;2(1):74-98.e9. doi: 10.1016/j.medj.2020.07.001 33511375 PMC7839961

[59] Kim HJ, Li H, Collins JJ, Ingber DE. Contributions of microbiome and mechanical deformation to intestinal bacterial overgrowth and inflammation in a human gut-on-a-chip. Proc Natl Acad Sci U S A. 2016;113(1):E7-15. doi: 10.1073/pnas.1522193112 26668389 PMC4711860

[60] Boquet-Pujadas A, Feaugas T, Petracchini A, Grassart A, Mary H, Manich M, et al. 4D live imaging and computational modeling of a functional gut-on-a-chip evaluate how peristalsis facilitates enteric pathogen invasion. Sci Adv. 2022;8(42):eabo5767. doi: 10.1126/sciadv.abo5767 36269830 PMC9586479

[61] Bein A, Kim S, Goyal G, Cao W, Fadel C, Naziripour A, et al. Enteric coronavirus infection and treatment modeled with an immunocompetent human intestine-on-a-chip. Front Pharmacol. 2021;12:718484. doi: 10.3389/fphar.2021.718484 34759819 PMC8573067

[62] Gunasekera S, Thierry B, King B, Monis P, Carr JM, Chopra A, et al. Microphysiological gut-on-chip enables extended in vitro development of *Cryptosporidium hominis*. Front Cell Infect Microbiol. 2025;15:1564806. doi: 10.3389/fcimb.2025.1564806 40343058 PMC12058726

[63] Sunuwar L, Yin J, Kasendra M, Karalis K, Kaper J, Fleckenstein J, et al. Mechanical stimuli affect *Escherichia coli* heat-stable enterotoxin-cyclic GMP signaling in a human enteroid intestine-chip model. Infect Immun. 2020;88(3):e00866-19. doi: 10.1128/IAI.00866-19 31818966 PMC7035931

[64] Bang LL, Pettersen JS, Høiland N, Rojek AM, Tornby DR, Møller-Jensen J, et al. An anaerobic in vitro flow model for studying interactions at the gastrointestinal host-microbe interface. NPJ Biofilms Microbiomes. 2025;11(1):160. doi: 10.1038/s41522-025-00800-z 40789857 PMC12340051

[65] Gazzaniga FS, Camacho DM, Wu M, Silva Palazzo MF, Dinis ALM, Grafton FN, et al. Harnessing colon chip technology to identify commensal bacteria that promote host tolerance to infection. Front Cell Infect Microbiol. 2021;11:638014. doi: 10.3389/fcimb.2021.638014 33777849 PMC7996096

[66] Lee J, Menon N, Lim CT. Dissecting gut‐microbial community interactions using a gut microbiome‐on‐a‐chip. Adv Sci. 2024;11:2302113. doi: 10.1002/advs.202302113 PMC11132043 38414327

[67] Penarete-Acosta D, Stading R, Emerson L, Horn M, Chakraborty S, Han A, et al. A microfluidic co-culture model for investigating colonocytes-microbiota interactions in colorectal cancer. Lab Chip. 2024;24(15):3690–703. doi: 10.1039/d4lc00013g 38973701

[68] Xu D, Guo M, Xu X, Luo G, Liu Y, Bush SJ, et al. Shigella infection is facilitated by interaction of human enteric α-defensin 5 with colonic epithelial receptor P2Y11. Nat Microbiol. 2025;10(2):509–26. doi: 10.1038/s41564-024-01901-9 39901059

[69] Yuan L, Haan P de, Peterson BW, Jong ED, Verpoorte E, Mei HC. Visualization of bacterial colonization and cellular layers in a gut-on-a-chip system using optical coherence tomography. Microsc Microanal. 2020;26:1211–9. doi: 10.1017/s143192762002454x 33107427

[70] Zhao W, Yao Y, Zhang T, Lu H, Zhang X, Zhao L, et al. Primary exploration of host-microorganism interaction and enteritis treatment with an embedded membrane microfluidic chip of the human intestinal-vascular microsystem. Front Bioeng Biotechnol. 2022;10:1035647. doi: 10.3389/fbioe.2022.1035647 36561041 PMC9763581

[71] Wu J, Fu K, Hou C, Wang Y, Ji C, Xue F, et al. Bacteriophage defends murine gut from *Escherichia coli* invasion via mucosal adherence. Nat Commun. 2024;15(1):4764. doi: 10.1038/s41467-024-48560-2 38834561 PMC11150434

[72] Mathew L, Carlson S, Savage M, Pardieu C, O’Brien M, Crawley E. Bacterial suppression of intestinal fungi via activation of human gut γδ T-cells. bioRxiv. 2025. doi: 10.1101/2025.09.17.676560

[73] Maurer M, Gresnigt MS, Last A, Wollny T, Berlinghof F, Pospich R, et al. A three-dimensional immunocompetent intestine-on-chip model as in vitro platform for functional and microbial interaction studies. Biomaterials. 2019;220:119396. doi: 10.1016/j.biomaterials.2019.119396 31398556

[74] Wang Y, Kim R, Sims CE, Allbritton NL. Building a thick mucus hydrogel layer to improve the physiological relevance of in vitro primary colonic epithelial models. Cell Mol Gastroenterol Hepatol. 2019;8: 653–5. doi: 10.1016/j.jcmgh.2019.07.009 PMC6889783 31356887

[75] Furtado KL, Plott L, Markovetz M, Powers D, Wang H, Hill DB, et al. *Clostridioides difficile*-mucus interactions encompass shifts in gene expression, metabolism, and biofilm formation. mSphere. 2024;9(6):e0008124. doi: 10.1128/msphere.00081-24 38837404 PMC11332178

[76] Duque-Correa MA, Goulding D, Rodgers FH, Gillis JA, Cormie C, Rawlinson KA, et al. Defining the early stages of intestinal colonisation by whipworms. Nat Commun. 2022;13(1):1725. doi: 10.1038/s41467-022-29334-0 35365634 PMC8976045

[77] Andronicos NM, McNally J, Kotze AC, Hunt PW, Ingham A. Trichostrongylus colubriformis larvae induce necrosis and release of IL33 from intestinal epithelial cells in vitro: Implications for gastrointestinal nematode vaccine design. Int J Parasitol. 2012;42(3):295–304. doi: 10.1016/j.ijpara.2012.01.007 22366550

[78] Ingle H, Hassan E, Gawron J, Mihi B, Li Y, Kennedy EA, et al. Murine astrovirus tropism for goblet cells and enterocytes facilitates an IFN-λ response in vivo and in enteroid cultures. Mucosal Immunol. 2021;14(3):751–61. doi: 10.1038/s41385-021-00387-6 33674763 PMC8085034

[79] Strine MS, Alfajaro MM, Graziano VR, Song J, Hsieh LL, Hill R, et al. Tuft-cell-intrinsic and -extrinsic mediators of norovirus tropism regulate viral immunity. Cell Rep. 2022;41(6):111593. doi: 10.1016/j.celrep.2022.111593 36351394 PMC9662704

[80] Greigert V, Saraav I, Son J, Zhu Y, Dayao D, Antia A, et al. Cryptosporidium infection of human small intestinal epithelial cells induces type III interferon and impairs infectivity of Rotavirus. Gut Microbes. 2024;16(1):2297897. doi: 10.1080/19490976.2023.2297897 38189373 PMC10793699

[81] Herzog MK-M, Cazzaniga M, Peters A, Shayya N, Beldi L, Hapfelmeier S, et al. Mouse models for bacterial enteropathogen infections: Insights into the role of colonization resistance. Gut Microbes. 2023;15(1):2172667. doi: 10.1080/19490976.2023.2172667 36794831 PMC9980611

[82] Fachi JL, Vinolo MAR, Colonna M. Reviewing the *Clostridioides difficile* mouse model: Insights into infection mechanisms. Microorganisms. 2024;12(2):273. doi: 10.3390/microorganisms12020273 38399676 PMC10891951

[83] Gilliland A, Chan JJ, De Wolfe TJ, Yang H, Vallance BA. Pathobionts in inflammatory bowel disease: Origins, underlying mechanisms, and implications for clinical care. Gastroenterology. 2024;166(1):44–58. doi: 10.1053/j.gastro.2023.09.019 37734419

[84] Thambugala KM, Daranagama DA, Tennakoon DS, Jayatunga DPW, Hongsanan S, Xie N. Humans vs. fungi: An overview of fungal pathogens against humans. Pathogens. 2024;13:426. doi: 10.3390/pathogens13050426 PMC11124197 38787278

[85] García FL, Leng J, Oliveira H, Nassoy P, Dementhon K. Collagen-based gut-on-chip for in vitro modeling of intestinal barrier function and host-pathogen interactions. bioRxiv. 2025. doi: 10.1101/2025.05.24.655930

[86] Alonso-Monge R, Gresnigt MS, Román E, Hube B, Pla J. *Candida albicans* colonization of the gastrointestinal tract: A double-edged sword. PLoS Pathog. 2021;17(7):e1009710. doi: 10.1371/journal.ppat.1009710 34293071 PMC8297749

[87] Gunasekera S, Thierry B, Cheah E, King B, Monis P, Carr JM, et al. A pumpless and tubeless microfluidic device enables extended in vitro development of *Cryptosporidium parvum*. Open Forum Infect Dis. 2024;11(11):ofae625. doi: 10.1093/ofid/ofae625 39512424 PMC11542632

[88] Nikolaev M, Mitrofanova O, Broguiere N, Geraldo S, Dutta D, Tabata Y, et al. Homeostatic mini-intestines through scaffold-guided organoid morphogenesis. Nature. 2020;585(7826):574–8. doi: 10.1038/s41586-020-2724-8 32939089

[89] Duque-Correa MA, Goulding D, Rodgers FH, Cormie C, Rawlinson K, Gillis JA. Defining the early stages of intestinal colonisation by whipworms. bioRxiv. 2021:2020.08.21.261586. doi: 10.1101/2020.08.21.261586 PMC8976045 35365634

[90] Hofer M, Duque-Correa MA, Lutolf MP. Patterned gastrointestinal monolayers with bilateral access as observable models of parasite gut infection. Nat Biomed Eng. 2025;9(7):1075–85. doi: 10.1038/s41551-024-01313-4 39633029 PMC7617323

[91] Lingappan K, Karmouty-Quintana H, Davies J, Akkanti B, Harting MT. Understanding the age divide in COVID-19: Why are children overwhelmingly spared? Am J Physiol Lung Cell Mol Physiol. 2020;319(1):L39–44. doi: 10.1152/ajplung.00183.2020 32491949 PMC7324935

[92] McGrath CJ, Laveckis E, Bell A, Crost E, Juge N, Schüller S. Development of a novel human intestinal model to elucidate the effect of anaerobic commensals on *Escherichia coli* infection. Dis Model Mech. 2022;15(4):dmm049365. doi: 10.1242/dmm.049365 35302159 PMC9066490

[93] Klasvogt S, Zuschratter W, Schmidt A, Kröber A, Vorwerk S, Wolter R, et al. Air-liquid interface enhances oxidative phosphorylation in intestinal epithelial cell line IPEC-J2. Cell Death Discov. 2017;3:17001. doi: 10.1038/cddiscovery.2017.1 28250970 PMC5327501

[94] Cai PC, Braunreuther M, Shih A, Spakowitz AJ, Fuller GG, Heilshorn SC. Air-liquid intestinal cell culture allows in situ rheological characterization of intestinal mucus. APL Bioeng. 2024;8(2):026112. doi: 10.1063/5.0187974 38721267 PMC11078553

[95] Charoensri T, Kulthong K, Pormrungruang P, Wongwanakul R, Kunyanee C, Aueviriyavit S, et al. Enterocyte culture on a hybrid transwell-inserted gut-on-a-chip for liquid-liquid and air-liquid interface conditions. ACS Omega. 2025;10(45):53865–73. doi: 10.1021/acsomega.5c03215 41280752 PMC12631310

[96] Finkbeiner SR, Hill DR, Altheim CH, Dedhia PH, Taylor MJ, Tsai Y-H, et al. Transcriptome-wide analysis reveals hallmarks of human intestine development and maturation in vitro and in vivo. Stem Cell Rep. 2015;4: 1140–55. doi: 10.1016/j.stemcr.2015.04.010 PMC4471827 26050928

[97] Aurora M, Spence JR. hPSC-derived lung and intestinal organoids as models of human fetal tissue. Dev Biol. 2016;420(2):230–8. doi: 10.1016/j.ydbio.2016.06.006 27287882 PMC5140713

[98] Kaiser KN, Snyder JR, Koppes RA, Koppes AN. A pumpless, high-throughput microphysiological system to mimic enteric innervation of duodenal epithelium and the impact on barrier function. Adv Funct Mater. 2024;34(51):2409718. doi: 10.1002/adfm.202409718 40873432 PMC12380100

[99] Kim HJ, Ingber DE. Gut-on-a-Chip microenvironment induces human intestinal cells to undergo villus differentiation. Integr Biol (Camb). 2013;5(9):1130–40. doi: 10.1039/c3ib40126j 23817533

[100] Mahapatro M, Foersch S, Hefele M, He G-W, Giner-Ventura E, Mchedlidze T, et al. Programming of intestinal epithelial differentiation by IL-33 derived from pericryptal fibroblasts in response to systemic infection. Cell Rep. 2016;15(8):1743–56. doi: 10.1016/j.celrep.2016.04.049 27184849

[101] Santos AJM, van Unen V, Lin Z, Chirieleison SM, Ha N, Batish A, et al. A human autoimmune organoid model reveals IL-7 function in coeliac disease. Nature. 2024;632(8024):401–10. doi: 10.1038/s41586-024-07716-2 39048815 PMC11747932

[102] Seiler KM, Bajinting A, Alvarado DM, Traore MA, Binkley MM, Goo WH, et al. Patient-derived small intestinal myofibroblasts direct perfused, physiologically responsive capillary development in a microfluidic Gut-on-a-Chip Model. Sci Rep. 2020;10(1):3842. doi: 10.1038/s41598-020-60672-5 32123209 PMC7051952

[103] Verhulsel M, Simon A, Bernheim-Dennery M, Gannavarapu VR, Gérémie L, Ferraro D, et al. Developing an advanced gut on chip model enabling the study of epithelial cell/fibroblast interactions. Lab Chip. 2021;21(2):365–77. doi: 10.1039/d0lc00672f 33306083 PMC9930731

[104] Jarret A, Jackson R, Duizer C, Healy ME, Zhao J, Rone JM. Enteric nervous system-derived IL-18 orchestrates mucosal barrier immunity. Cell. 2020;180:50–63. doi: 10.1016/j.cell.2019.12.016 PMC7339937 31923399

[105] Costa DVS, Thomasi B, Brito GAC, Gulbransen BD, Warren CA. The role of the enteric nervous system in the pathogenesis of *Clostridioides difficile* infection. Nat Rev Gastroenterol Hepatol. 2025;22(8):570–86. doi: 10.1038/s41575-025-01071-x 40404838 PMC12889098

[106] Manion J, Musser MA, Kuziel GA, Liu M, Shepherd A, Wang S, et al. C. difficile intoxicates neurons and pericytes to drive neurogenic inflammation. Nature. 2023;622(7983):611–8. doi: 10.1038/s41586-023-06607-2 37699522 PMC11188852

[107] Sedrani C, Gomez-Giro G, Grandmougin L, Schwamborn JC, Wilmes P. A gut-on-a-chip model to study the gut microbiome-nervous system axis. J Vis Exp. 2023;(197):10.3791/64483. doi: 10.3791/64483 37578222

[108] Wang H, Foong JPP, Harris NL, Bornstein JC. Enteric neuroimmune interactions coordinate intestinal responses in health and disease. Mucosal Immunol. 2022;15(1):27–39. doi: 10.1038/s41385-021-00443-1 34471248 PMC8732275

[109] Izadifar Z, Charrez B, Almeida M, Robben S, Pilobello K, van der Graaf-Mas J, et al. Organ chips with integrated multifunctional sensors enable continuous metabolic monitoring at controlled oxygen levels. Biosens Bioelectron. 2024;265:116683. doi: 10.1016/j.bios.2024.116683 39213819 PMC11391946

[110] Shi H, Kowalczewski A, Vu D, Liu X, Salekin A, Yang H, et al. Organoid intelligence: Integration of organoid technology and artificial intelligence in the new era of in vitro models. Med Nov Technol Devices. 2024;21:100276. doi: 10.1016/j.medntd.2023.100276 38646471 PMC11027187

[111] Hou K, Wu Z-X, Chen X-Y, Wang J-Q, Zhang D, Xiao C, et al. Microbiota in health and diseases. Signal Transduct Target Ther. 2022;7(1):135. doi: 10.1038/s41392-022-00974-4 35461318 PMC9034083

[112] Lee Y, Kim M-H, Alves DR, Kim S, Lee LP, Sung JH, et al. Gut-kidney axis on chip for studying effects of antibiotics on risk of hemolytic uremic syndrome by shiga toxin-producing *Escherichia coli*. Toxins (Basel). 2021;13(11):775. doi: 10.3390/toxins13110775 34822559 PMC8622205

[113] Delannoy E, Burette A, Janel S, Poiret S, Deboosere N, Daniel C, et al. Gut-on-chip methodology based on 3D-printed molds: a cost-effective and accessible approach. Lab Chip. 2025;25(17):4396–409. doi: 10.1039/d5lc00147a 40689563

[114] Morelli M, Cabezuelo Rodríguez M, Queiroz K. A high-throughput gut-on-chip platform to study the epithelial responses to enterotoxins. Sci Rep. 2024;14(1):5797. doi: 10.1038/s41598-024-56520-5 38461178 PMC10925042

[115] Criss ZK 2nd, Bhasin N, Di Rienzi SC, Rajan A, Deans-Fielder K, Swaminathan G, et al. Drivers of transcriptional variance in human intestinal epithelial organoids. Physiol Genomics. 2021;53(11):486–508. doi: 10.1152/physiolgenomics.00061.2021 34612061 PMC8616596

[116] Pike CM, Zwarycz B, McQueen BE, Castillo M, Barron C, Morowitz JM, et al. Characterization and optimization of variability in a human colonic epithelium culture model. ALTEX. 2024;41(3):425–38. doi: 10.14573/altex.2309221 38641922 PMC12713543

[117] Kim D, Youn J, Kim J, Lee J, Yoon J, Kim DS. From organoid culture to manufacturing: Technologies for reproducible and scalable organoid production. NPJ Biomed Innov. 2026;3(1):12. doi: 10.1038/s44385-025-00054-6 42031991 PMC13042217

[118] Sieders M, Candry P, El Aidy S. Hydrogel-based experimental models of the gastrointestinal tract. Microbiome. 2025;13(1):233. doi: 10.1186/s40168-025-02208-5 41233829 PMC12613474

[119] Xiang X, Wang X, Shang Y, Ding Y. Microfluidic intestine-on-a-chip: Current progress and further perspectives of probiotic-foodborne pathogen interactions. Trend Food Sci Tech. 2023;134:207–21. doi: 10.1016/j.tifs.2023.03.006

[120] Fu K, Cui J, Li Y, Zhang Y, Wang Y, Wu J, et al. *Escherichia coli* phage ΦPNJ-9 adheres to mucus via a variant Hoc protein. J Virol. 2025;99(2):e0178924. doi: 10.1128/jvi.01789-24 39723818 PMC11853027

[121] Choi K-YG, Wu BC, Lee AH-Y, Baquir B, Hancock REW. Utilizing organoid and air-liquid interface models as a screening method in the development of new host defense peptides. Front Cell Infect Microbiol. 2020;10: 228. doi: 10.3389/fcimb.2020.00228 PMC7251080 32509598

